# Investigating the Impact of Indemnity Waivers on the Length of Stay of Cats at an Australian Shelter

**DOI:** 10.3390/ani9020050

**Published:** 2019-02-07

**Authors:** Jessica Pockett, Bronwyn Orr, Evelyn Hall, Wye Li Chong, Mark Westman

**Affiliations:** 1 Sydney School of Veterinary Science, The University of Sydney, Camperdown, NSW 2006, Australia; jpoc8742@uni.sydney.edu.au (J.P.); mark.westman@sydney.edu.au (M.W.); 2 RSPCA ACT, Weston, ACT 2611, Australia; wyechong@gmail.com; 3 Sydney School of Veterinary Science, The University of Sydney, Camden, NSW 2570, Australia; evelyn.hall@sydney.edu.au

**Keywords:** cat, shelter, RSPCA, length of stay, indemnity waivers

## Abstract

**Simple Summary:**

The practice of adopting animals from shelters with ‘indemnity waivers’ is becoming increasingly common. Indemnity waivers serve to limit the ongoing responsibility of a shelter to an animal with a pre-existing condition likely to involve veterinary treatment in the future, thereby allowing shelters to adopt out animals that may have been previously considered unsuitable for adoption and instead euthanased. However, there has been concern from some sectors of the sheltering community that indemnity waivers can lead to animals staying in shelters longer than necessary because with a waiver they become less desirable to the public. This research sought to examine if there was a link between the presence of indemnity waivers and increased lengths of stay (LOS) of cats at an Australian animal shelter. It examined data for 249 cats adopted from the Royal Society for the Prevention of Cruelty to Animals (RSPCA) Weston shelter located in the Australian Capital Territory (ACT), Australia over a period of six months in 2017. The results demonstrated that cats adopted with indemnity waivers were found to have a longer LOS than those adopted without waivers, however no particular waiver type was found to be responsible for this effect. This finding should encourage shelters to use indemnity waivers judiciously due to the impact on LOS.

**Abstract:**

Due to resource limitations, animal shelters in Australia historically have focused on rehoming animals considered ‘highly adoptable’. Increasingly, animal shelters in Australia are rehoming animals with pre-existing medical and/or behavioural issues. These animals are often rehomed with an ‘indemnity waiver’ to transfer the responsibility of ongoing financial costs associated with these conditions from the shelter to the new owner. However, it is unknown what effect these indemnity waivers have on the length of stay (LOS) of animals prior to adoption. The current study used data collected from the Royal Society for the Prevention of Cruelty to Animals (RSPCA) Weston shelter located in the Australian Capital Territory (ACT), Australia in 2017 to investigate the effect of indemnity waivers on the LOS of cats. A restricted maximum likelihood model (REML) was used to determine the effect of breed, age, coat colour, presence of a waiver, waiver type (categorised into seven groups) and waiver number (no waiver, single waiver or multiple waivers) on LOS. In the final multivariate model, age, breed and waiver number were found to influence LOS. Young cats, purebred cats and cats adopted without a waiver were adopted fastest. This study is the first to report the effect of indemnity waivers on the adoptability of cats from shelters.

## 1. Introduction

Animal shelters play a vital role in providing a safe and comfortable environment for unwanted, stray and injured animals before they are reunited, rehabilitated, rehomed or, as a last resort, euthanased [1,2]. One organisation that works to care for and protect animals in Australia, the Royal Society for the Prevention of Cruelty to Animals (RSPCA), receives thousands of animals annually in every state and territory [3]. During the 2016–2017 financial year, RSPCA Australia received a total of 135,872 animals, with cats comprising the highest proportion (53,912; 40%) of the total number of animals received [1]. The majority of these cats (33,253; 62%) were either rehomed or reclaimed, but a proportion (14,563; 27%) were euthanased for a variety of reasons [2].

The aim of every rehoming organisation is to maximise rehoming and minimise euthanasia. Integral to both of these aims is to minimise the length of stay (LOS) of animals (defined as the amount of time from availability to adoption). The longer an animal’s LOS, the higher the risk becomes of that animal contracting disease (in particular, feline upper respiratory tract infection and dermatophytosis in cats) or developing negative behavioural traits [3,4]. This then creates a cycle whereby the animal becomes less adoptable, the LOS is increased further, and euthanasia becomes more likely [2,3,5,6,7]. Animals with a prolonged LOS also occupy valuable cage space which might be better used to rehome more adoptable animals. Therefore, LOS is an important statistic that is monitored closely by shelters, and resources are targeted towards strategies to reduce LOS.

A cat’s behaviour, temperament and personality have been found to be important factors influencing potential adopters’ choices when selecting a cat, in particular playfulness and approachability, even more than physical appearance [8,9,10,11,12,13]. Physical traits that are associated with a shorter LOS include age (younger cats), sex (male), breed (exotic/purebred), coat colour (light-coloured) and coat pattern (tortoiseshell–tabby) [8,13,14].

In response to changing community expectations about acceptable euthanasia rates, shelters are increasingly rehoming animals with pre-existing medical or behavioural issues to new owners who sign an ‘indemnity waiver/s’ at the time of adoption. An indemnity waiver is a legal document to ensure that the new owner understands the animal is being rehomed with a pre-existing condition/s, and that all financial costs associated with the pre-existing condition/s will become the responsibility of the new owner following adoption. However, the effect of indemnity waivers on LOS and adoptability is unknown. It is assumed by shelters that animals without pre-existing conditions are generally rehomed faster than those animals rehomed with identified conditions, due to the additional time and financial costs possibly required of a new owner.

The aim of this study was to determine the effect of indemnity waivers on the LOS of cats rehomed from an Australian shelter.

## 2. Materials and Methods

### 2.1. Study Population

This study used computerised adoption records from cats admitted to a managed RSPCA admission shelter in Weston, Australian Capital Territory (ACT), Australia. Cats were either owner-relinquished or strays. Once admitted to the shelter, a cat underwent a full physical examination by a shelter veterinarian, was vaccinated with a core trivalent vaccine (F3), treated for internal and external parasites, and, if needed, microchipped. Any obvious medical abnormalities were noted at this time by the veterinarian and, if no further intervention was required, an indemnity waiver was placed on the cat’s file to inform new owners of any medical issues that required consideration prior to adoption. A behavioural examination was then performed by a senior shelter staff member trained in behavioural assessments and, based on this assessment, the cat was either passed as being behaviourally suitable for adoption and transferred to a cage in the rehoming area, or was left to settle in a holding cage. If the cat was left to settle, a further behavioural examination was performed three days later. Any perceived behavioural issues observed during either examination were recorded as an indemnity waiver. Once a surrendered cat had passed both its medical and behavioural assessment, if it did not require any veterinary treatment, it was immediately ready for adoption. Stray cats were required to complete a mandatory holding period of seven days before ownership was assigned to the RSPCA and they could be made available for adoption. Surrendered and stray cats that required additional tests and procedures, such as desexing, dental procedures, radiographs and surgery, had these interventions performed before they became available for adoption.

### 2.2. Description of Data Set

All cats six months of age and older adopted between the 1st of June and the 11th of December 2017 were included for analysis. Cats under six months of age were excluded as cats in this age bracket are rarely rehomed with any type of waiver from the shelter (W. Chong, *per comms*). In addition, young cats (<6 months) are generally rehomed faster than adult cats, meaning this group would not have represented a suitable non-waiver group for comparison. Three cats rehomed with a ‘geriatric waiver’ were excluded from final analysis, since this waiver may have been a confounding factor when considering the effect of age on LOS. These geriatric cats had no pre-existing conditions identified on physical examination, and the geriatric waiver was simply seen a way of fast-tracking adoption without having to perform additional testing, e.g., biochemistry testingfor chronic kidney disease or hyperthyroidism (W. Chong, *per comms*). Cats not placed for adoption, such as those reclaimed by owners or euthanased for medical or behavioural reasons, were also excluded from the study.

Information collected for the purpose of this study included the cat’s identification number, sex, estimated primary breed, primary coat colour/pattern, approximate or known age, description of indemnity waiver/s, number of waivers (if more than one), reason for return (if previously returned) and LOS. Data were extracted from the shelter management software (Shelter Buddy^®^) and exported to and managed in Microsoft Excel^®^.

### 2.3. Age Categories

Age was estimated by RSPCA employees based on physical traits including dental wear. Based on this estimate, ‘juveniles’ were defined as cats aged 6 months to 1 year, ‘young adults’ 1–5 years, ‘adults’ 5–8 years and ‘geriatric adults’ 8 years and older.

### 2.4. Breed Designations

Cats were categorised into primary breeds based on phenotype. From an original characterisation of 13 breeds, breed was retrospectively standardised by the primary author (JP) into two groups for study analysis: ‘Non-purebred’, which included domestic short-haired, domestic medium-haired, domestic long-haired and domestic bob-tailed cats; and ‘Purebred’, which included Siamese, Tonkinese, Burmese, Russian Blue, Birman, Ragdoll, Bengal cross, Turkish Van and Abyssinian cats.

### 2.5. Coat Colour Definitions

Coat colour was retrospectively categorised into seven groups by the primary author (JP) to facilitate statistical analysis. The final seven groups, with the original coat colour included in parentheses, were: Dark (black, brown and chocolate), ginger, white, grey (grey, blue, lilac), two tone (black/white, brown/white, grey/white, blue/white, ginger/white, tabby/white, lilac point/seal point), tortoiseshell and tabby.

### 2.6. Length of Stay (LOS)

LOS was defined as the time (in whole days) from when the animal’s status on Shelter Buddy^®^ was changed from ‘available for adoption’ to ‘adopted’. The calculated LOS did not include the mandatory holding period for stray cats or the time spent awaiting initial examination, routine testing, behavioural assessment or treatment by a veterinarian. As some cats were made immediately available for adoption and others had to remain for a holding period, LOS was defined as a conservative variable for statistical analysis. For cats that were returned to the shelter after being adopted, only their first LOS was included in the analysis. If returned cats were subsequently euthanased, their data were still included in the analysis.

### 2.7. Waiver Type and Waiver Number

A brief description of individual waivers (*n* = 26) is provided in Table 1, with these waivers retrospectively categorised into seven groups (‘waiver type’) by the primary author (JP) for analysis (Table 2). For analysis, both waiver type and the number of waivers an individual cat held (0, 1 and 2+) were considered. Cats rehomed with two or more waivers were considered together to make the waiver number group sizes comparable (Table 3).

### 2.8. Returned Cats

Cats were also split into two groups (later returned versus not returned) for bimodal analysis. 

### 2.9. Statistical Analysis

An Anderson Darling test for normality determined that the LOS data were not normally distributed, and examination of a Q–Q plot demonstrated that logarithmic transformation was suitable. All statistical analysis was conducted in GenStat^®^ (v.17, VSNi). For analyses, a *p* value of < 0.05 was considered significant. As some cats were adopted within 24 h of entering the shelter and had a ‘0’ LOS, each LOShad 1 added to its value to make the data statistically meaningful. Two separate Restricted Maximum Likelihood (REML) models were used: Firstly, the impact of age, breed, colour, waiver presence (Y/N) and waiver number (0, 1, 2+) on LOS; and secondly, the impact of waiver type on LOS. Univariate analyses were conducted on each model to determine initial significance, followed by a stepwise backwards elimination approach to determine a final model where all factors were significant. Post-hoc testing was conducted using least significant differences (LSD) to determine significant pairwise comparisons for significant effects. Bimodal analysis was conducted to determine if there was a significant difference in LOS between cats that were returned and cats that were not returned.

### 2.10. Ethics Approval

Ethics approval was not sought for this study. The research presented in this paper is analysis of historical data involving an accepted practice, i.e., applying indemnity waivers to animals available for adoption. No animals or humans were directly involved or recruited for the study. Written permission was received from the shelter supplying the data for analysis before research commenced.

## 3. Results

### 3.1. Study Population

The final study cohort comprised of 249 cats of varying ages, sexes, breeds and health statuses (Table 3). The average LOS for the 249 cats rehomed during the study period was 8.8 days, with a range of less than 1 day to 70 days.

### 3.2. Univariate Analysis

**Figure 1:**
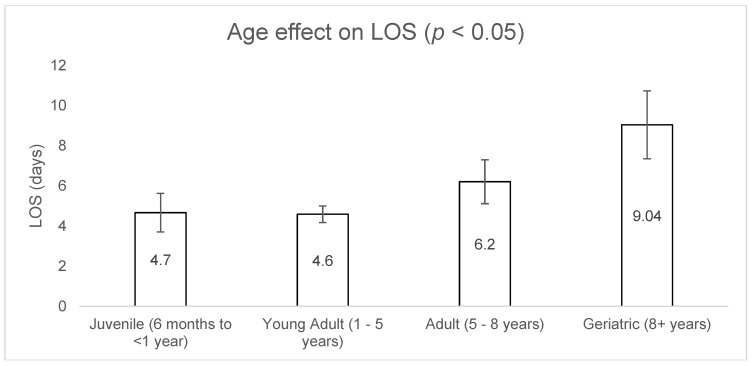
Effect of age on LOS. Age was found to have a significant effect, with juvenile and young adult cats rehomed faster than adult and geriatric cats (*p* = 0.009).

Controlling for other variables (breed, age, coat colour, waiver presence and waiver type), sex was not found to have a significant effect on LOS (females 5.6 ± 0.53 days versus males 4.8 ± 0.50 days; *p* = 0.312). Age was found to have a significant effect on LOS, with younger cats generally adopted fastest (*p* = 0.009). Geriatric cats were found to have the longest LOS, remaining in the shelter for an average of 9 ± 1.69 days, followed by adult cats (6.2 ± 1.09 days), juvenile cats (4.7 ± 0.96 days) and young adults (4.6 ± 0.41 days) (Figure 1). Purebred cats had a 62% lower mean LOS compared to non-purebred cats (2.1 ± 0.6 days versus 5.5 ± 0.39 days; *p* < 0.001). Coat colour was not found to have a significant effect on LOS (*p* = 0.476). Cats rehomed with an indemnity waiver/s had a significantly higher mean LOS compared to the non-waiver population (5.9 ± 0.51 days versus 4.1 ± 0.49 days; *p* = 0.015). Waiver number was also found to have a significant effect on LOS (*p* = 0.006). Cats that were rehomed without a waiver had the shortest LOS (4.15 ± 0.48 days), followed by cats rehomed with one waiver (5.1 ± 0.56 days) and cats rehomed with multiple waivers (7.41 ± 1.02 days). A summary of these results is presented in Table 4.

### 3.3. Multivariate Analysis

**Figure 2:**
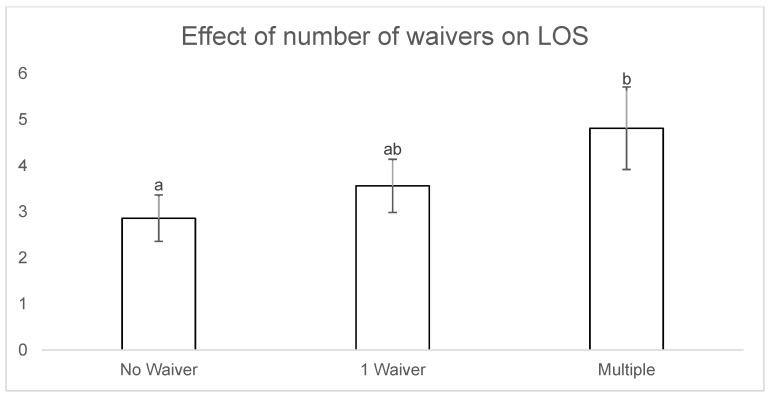
Effect of waiver number on LOS.

Age, breed and waiver number were included in the REML multivariable model to assess the effects of these proposed predictor values on LOS, based on significant results from univariate analysis (*p* < 0.05). In the multivariate model, age, breed and waiver number remained significant (*p* = 0.004, *p* < 0.001 and *p* = 0.016, respectively) (Table 5). There was a significantly increased LOS for cats rehomed with multiple waivers compared to cats rehomed without a waiver (4.8 ± 0.89 days versus 2.8 ± 0.5 days; *p* = 0.016) (Figure 2). Possible interactions between breed and age, age and waiver number and waiver number and breed were then tested, with no interactions observed (*p* = 0.83, *p* = 0.23 and *p* = 0.45, respectively).

**Figure 3:**
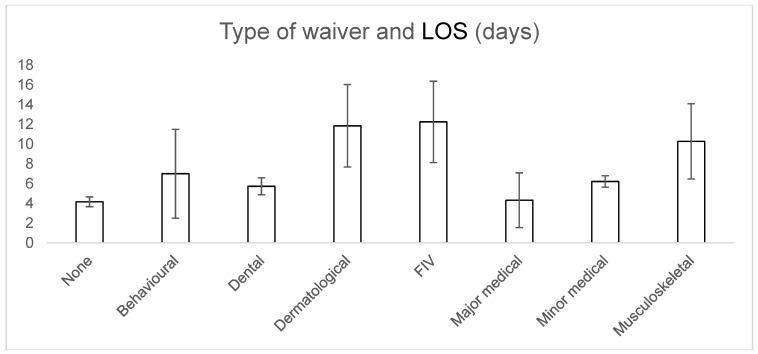
Effect of waiver type on LOS.

With relation to waiver type, none were found to be significant when compared to the average LOS for the waiver group (Figure 3): Behavioural (7.0 ± 4.5 days, *p* = 0.98), FIV (12.2 ± 4.1 days, *p* = 0.062), major medical (4.3 ± 2.8 days, *p* = 0.32), minor medical (6.2 ± 0.6 days, *p* = 0.28), musculoskeletal (10.3 ± 3.8 days, *p* = 0.23), dental (5.7 ± 0.6 days, *p* = 0.54) and dermatological (*p* = 0.093).

### 3.4. Bimodal Analysis

There was no significant difference in LOS between cats that were later returned (*n* = 20) and those that were not returned (6.8 ± 4.17 days versus no return 5.8 ± 1.07 days; *p* = 0.654, returned).

## 4. Discussion

The adoptability of cats from an Australian shelter, as measured by LOS, was influenced by age, breed, the presence of an indemnity waiver and the number of indemnity waivers. This is, to our knowledge, the first time the effect of pre-existing conditions on adoptability has been reported in the literature. Cats rehomed with an indemnity waiver/s were found to have a significantly longer LOS compared to cats rehomed without a waiver. This finding was most likely the result of waivers being a major point of discussion and deliberation during the adoption process, with potential owners needing to consider if they had the time, capacity and finances to appropriately care for the particular requirements of the cat. Cats rehomed with multiple waivers took significantly longer to rehome than cats with a single waiver, suggesting that potential adopters were deterred by multiple issues. Waiver type was not found to have a significant effect on LOS. However, both the FIV and dermatological waiver groups had *p*-values that approached significance (*p* = 0.062 and 0.093, respectively). The small sample sizes for these groups (11 and 10 cats, respectively) may have contributed to this conclusion, and it is possible that significant associations may have been found with larger sample sizes, meaning more research in this area is needed.

Age was found to significantly affect LOS, with young adults having the shortest LOS, followed by juveniles, adults and geriatrics. A study conducted across three cat shelters in the Czech Republic reported that adult cats had the shortest LOS, followed by kittens, juveniles, young adults and geriatric cats [8]. Young adult cats may have had the shortest LOS in our study due to an overrepresentation (145/249 = 58%). Similar to the results from our study, geriatric cats rehomed from a no-kill shelter in New York State had the longest LOS [14], and other studies have reported that the likelihood of adoption decreased with increasing age [6,11,14,15,16]. It is clear that age is an important factor for predicting LOS, with increased age generally resulting in an increased LOS [13]. 

Breed was found to influence LOS in this study, with purebred cats having a shorter LOS compared to non-purebred cats. This finding is consistently reported in the literature [14,17]. One study concluded that Persians, Russian Blues and Ragdolls had a LOS 64% shorter than non-purebred cats, similar to the 62% reported in our study [2]. 

Sex was not found to have a significant effect on the LOS of cats in our study. This contrasts from the general trend reported in other studies, with males repeatedly found to be adopted faster than females [10,14,16]. Brown and Morgan (2015) reported that male kittens were adopted on average ten days faster than females [14], and similarly Janke et al. (2017) reported that males had a LOS 20% shorter than females [2]. It has been suggested that this finding is the result of behavioural differences between males and females, with male cats approaching potential adopters more readily than females and being more playful [14]. In contrast, a study conducted across three shelters in the Czech Republic found no difference in LOS between male and female cats [8]. However, this finding was thought to be a result of the predominance of female cats in the shelters [8]. As our study also had a slight predominance of female cats (133/249 = 56%), it is possible that our results were similarly skewed, or may have been affected by an insufficient sample size. 

Coat colour was another physical trait that was found to have no impact on LOS in the current study. This finding contrasts many other studies that have reported a significant impact of coat colour on LOS [8,14,16,17]. Generally, cats with darker coats have been reported to take longer to adopt than medium-shade or lighter coloured cats [8,14,16,17]. Kubesova et al. (2017) reported that darker coloured cats remained in shelters approximately one month longer than other coloured cats [8]. Similarly, Brown and Morgan (2015) reported that cats with seal colouration had the shortest LOS, whilst cats primarily yellow or black in colour had the longest LOS [14]. The difference in coat colour findings from our study compared to previous studies is most likely attributed to our small sample size and limited variation within each colour category. It is also possible that local preferences for coat colour and pattern exist among shelters, and this possibility should be explored further [15].

The finding that the presence of an FIV waiver did not have a significant impact on LOS is important as previously some shelters have not rehomed FIV-positive cats on the premise that they will have a prolonged LOS and occupy space that could be used for more adoptable cats [18]. In one unpublished study, 4/17 Australian shelters responded that they euthanased all FIV-positive cats regardless of health status (B. Orr, *per comms*). Results from this study may therefore increase the number of FIV-positive cats rehomed from shelters in Australia. With appropriate management, FIV-infected cats can live as long as FIV-uninfected cats and, if kept indoors, FIV-positive cats pose a minimal transmission risk to other cats [19].

Out of a total of 249 cats studied, only 20 cats were returned to the shelter. Out of these 20 cats, 7 had not been assigned a waiver. Bimodal analysis revealed that there was no significant difference found between cats that had been returned and those that had not. Reasons for return included both human and animal factors: behavioural or temperament related issues (most common), lack of time, moving to a new house, allergies to the animal, the animal not being house trained, owner health or ‘just because’. Further research should be conducted to determine if this finding is representative of return rates for other animal shelters. Additionally, it would be useful to determine the return rate of cats adopted with a waiver/s, and if the return rate differs by waiver type. 

Important variables that may impact cats’ LOS in shelters and were not considered in the current study include the behaviour of the animal, the photo of the animal used on the website and any associated text, provision of toys, time of year, the name of the cat, the description of the cat on the cage card, the relationship of the cat to adoption staff and the language adoption staff used to describe the cat, as well as any seasonal effects. Future LOS studies should attempt to take into consideration as many of these variables as possible. A cat’s behaviour has been found to be the primarily factor influencing potential adopters’ choice in selecting a cat, with temperament and personality ranked higher than physical appearance [9,10,11,12,13]. Based on a questionnaire, cats that appeared ‘friendly’, ‘happy’, ‘relaxed’, ‘playful’, ‘friendly with other cats’ and ‘smart’ were most attractive to potential adopters [12]. The same questionnaire revealed that individual cats and cats observed trying to hide in the litter box were unattractive to potential adopters [12]. In other studies, active cats and cats provided with toys in their cage were viewed for significantly longer than cats that were less active or without toys [11,12]. Future research, which was not possible in the current study due to the retrospective design, should attempt to investigate any potential correlation between LOS and whether a cat passed its initial behavioural examination or needed to undergo additional behavioural testing. Cat adoption has also been found to be influenced by season, with one study reporting that adoption rates increased following the breeding season [8]. Certain medical conditions are also more prevalent during specific periods of the year, for example flea allergy dermatitis (FAD) is usually worse during the summer months, which may result in an increased number of cats with dermatological waivers during this period. As only one cat in the current study presented with a seasonal condition (FAD), the effect of seasonal conditions on waiver type and LOS was not investigated. 

Finally, this study primarily assessed the impact of a waiver on cats’ LOS but did not assess whether or not this impact was the consequence of the waiver itself or the arrangement it resulted in. Further research to determine if the impact was the result of the waiver (and signing process), or the post-adoption conditions required by the waiver, would therefore be useful.

## 5. Conclusions

This study determined that the presence of a waiver, but not the waiver type, had a significant negative impact on the LOS of cats rehomed from an Australian shelter. Shelters should direct resources, reserve prominent cage locations and target media efforts towards rehoming cats with waivers in order to reduce LOS and increase rehoming rates. Shelters should also consider whether a waiver is absolutely necessary (e.g., for very minor conditions), knowing that it may increase the LOS of these cats.

## Figures and Tables

**Table 1:** Cat adoption waivers used at the RSPCA Weston shelter in the ACT, Australia with accompanying explanations for potential adopters.

Waiver	Explanation or Reason for Waiver
Obesity (*n* = 5)	Animal requires weight reduction program to return to a healthy weight.
Squamous cell carcinoma (SCC) risk (*n* = 95)	Risk of SCC due to white or pink features on face and ears.
‘Cat flu’ (*n* = 33)	Exposed or previously contracted cat flu (feline upper respiratory tract disease), likely to have latent viral infection for life.
Umbilical hernia (*n* = 1)	A condition in which intestines may possibly protrude through the abdominal muscles at the umbilicus.
FLUTD (feline lower urinary tract disease) (*n* = 7)	Showed evidence of blood in urine or FLUTD-like signs whilst in care, possibly predisposed to FLUTD in the future.
Ringworm (*n* = 5)	Exposed or previously contracted ringworm.
Flea allergy dermatitis (FAD) (*n* = 1)	Showed signs of FAD in care, owners will need to continue flea treatment to prevent recurrence.
Dental disease (*n* = 44)	Minor or major dental disease identified on examination, likely to require some treatment for dental disease in future (minor = scale and polish, major = extractions).
Teeth extracted (*n* = 11)	Major dental surgery performed with the possibility for future dental disease, similar to waiver ‘Dental disease’
Osteoarthritis (*n* = 6)	Evidence of arthritis, management of condition required in future.
Tremoring (*n* = 1)	Undiagnosed neurological tremor.
Abscess (*n* = 1)	Presented to shelter with an abscess which was in the process of healing.
FIV (feline immunodeficiency virus) (*n* = 12)	Tested FIV-positive, not showing clinical signs, disease may progress in future.
Feline acne (*n* = 3)	Evidence of feline acne whilst in care, management changes will be required in future.
Femoral head excision (FHE) (*n* = 1)	Femoral head was surgically removed in care due to previous trauma, ongoing management may be required.
Pelvic fractures (*n* = 1)	Old injury, likely will have arthritis in future.
Luxating patella (*n* = 1)	Diagnosed with a low grade luxating patella, not surgically corrected, may require ongoing management.
Hyperthyroidism (*n* = 1)	Surrendered on hyperthyroid medication, however no clinical signs while in care and T3 and T4 levels normal. May require treatment for hyperthyroidism in the future.
CKD (chronic kidney disease) (*n* = 1)	Diagnosed with CKD whilst in care, management of the condition will be required.
Oral trauma (*n* = 1)	Presented as such, still healing during adoption.
Corneal ulceration (*n* = 1)	Presented as such, still healing during adoption.
Anxiety (*n* = 1)	Will need ongoing management.
Skin mites (*n* = 1)	Diagnosed whilst in care and treated but might reoccur.
Pica (*n* = 1)	The persistent behaviour of eating non-food material (e.g., blankets, socks, grocery bags).
Medical fail (*n* = 1)	Chronic medical issue likely to deteriorate with time (e.g., advanced CKD, congenital heart disease).
Wound (*n* = 1)	Presented with large skin wound, still healing during adoption.

**Table 2:** Categorisation of waivers from Table 1 into seven groups (‘waiver type’) for the purposes of statistical analysis. The total number of waiver types included for this study is summarised in Table 3.

Waiver Type	Waivers Included
Behavioural	Pica, anxiety and tremoring
Feline immunodeficiency virus (FIV)	FIV-positive
Major medical	CKD, hyperthyroidism and medical fail
Minor medical	Abscess, umbilical hernia, obesity, ‘cat flu’, SCC risk, oral trauma, corneal ulceration, wound and FLUTD
Musculoskeletal	Luxating patella, FHE, pelvic fractures and osteoarthritis
Dermatological	Skin mites, FAD, feline acne and ringworm
Dental disease	Minor dental disease—without extractions; major dental disease—with extractions

**Table 3:** Summary of the study cohort (*n* = 249) by sex, breed, age, presence or absence of a waiver, number of waivers assigned to each cat (‘waiver number’) and waiver type. In total there were 237 waiver types analysed due to the variation in waiver number between cats (i.e., 0, 1 and 2+).

Variable	Categories	Number
Sex	Male	116
	Female	133
Breed	Non-purebred	234
	Purebred	15
Age	Juveniles (6–12 months)	28
	Young adults (1–5 years)	145
	Adults (5–8 years)	38
	Geriatrics (>8 years)	34
	Unknown	4
Coat colour	Dark	55
	Ginger	8
	White	11
	Grey	12
	Two tone	38
	Tortoiseshell	30
	Tabby	95
Waiver presence	Yes	163
	No	86
Waiver number	No waiver	88
	One waiver	99
	Multiple waivers (i.e., 2 or more) *	62
Waiver type	Behavioural	3
	Feline immunodeficiency virus (FIV)	11
	Major medical	3
	Minor medical	146
	Musculoskeletal	9
	Dermatological	10
	Dental disease	55

* Cats with two or more waivers were considered together to make the waiver number groups of comparable size. Multiple waivers included 50 cats with two waivers, 11 cats with three waivers and one cat rehomed with five waivers.

**Table 4:** REML univariate model output assessing interactions between predictor values and LOS.

Name of Variable	Wald Statistic	n.d.f	F Statistic	d.d.f	F pr
Sex	1.03	1	1.03	247.0	0.312
Breed	10.91	1	10.91	247.0	0.001
Age	11.95	3	3.98	241.0	0.009
Colour	5.56	6	0.93	242.0	0.476
Waiver (yes/no)	5.97	1	5.97	247.0	0.015
Total waiver number (0, 1, 2+)	10.39	2	5.19	246.0	0.006

**Table 5:** REML multivariate model output assessing interactions between predictor values and LOS (final model).

Name of Variable	Wald Statistic	n.d.f	F Statistic	d.d.f	F pr
Age	13.63	3	4.54	238.0	0.004
Breed	17.36	1	17.36	238.0	<0.001
Waiver number	8.41	2	4.20	238.0	0.016
